# The Causal Interaction between Complex Subsystems

**DOI:** 10.3390/e24010003

**Published:** 2021-12-21

**Authors:** X. San Liang

**Affiliations:** 1 Department of Atmospheric & Oceanic Sciences, Institute of Atmospheric Sciences, Fudan University, Shanghai 200438, China; xsliang@fudan.edu.cn; 2 IRDR ICoE on Risk Interconnectivity and Governance on Weather/Climate Extremes Impact and Public Health, Fudan University, Shanghai 200438, China; 3 Shanghai Qi Zhi Institute (Andrew C. Yao Institute for Artificial Intelligence), Shanghai 200232, China

**Keywords:** bulk information flow, complex system, causality, subspace, networks of networks

## Abstract

Information flow provides a natural measure for the causal interaction between dynamical events. This study extends our previous rigorous formalism of componentwise information flow to the *bulk* information flow between two complex subsystems of a large-dimensional parental system. Analytical formulas have been obtained in a closed form. Under a Gaussian assumption, their maximum likelihood estimators have also been obtained. These formulas have been validated using different subsystems with preset relations, and they yield causalities just as expected. On the contrary, the commonly used proxies for the characterization of subsystems, such as averages and principal components, generally do not work correctly. This study can help diagnose the emergence of patterns in complex systems and is expected to have applications in many real world problems in different disciplines such as climate science, fluid dynamics, neuroscience, financial economics, etc.

## 1. Introduction

When investigating the properties of a complex system, it is often necessary to study the interaction between one subsystem and another subsystem, which themselves also form complex systems, usually with a large number of components involved. In climate science, for example, there is much interest in understanding how one sector of the system collaborates with another sector to cause climate change (see [1] and the references therein); in neuroscience, it is important to investigate the effective connectivity from one brain region to another, each with millions of neurons involved (e.g., [2,3]), and the interaction between structures (e.g., [4,5,6]; see more references in a recent review [7]). This naturally raises a question: How can we study the interaction between two subsystems in a large parental system?

An immediate answer coming to mind might be to study the componentwise interactions by assessing the causalities between the respective components using, for instance, the classical causal inference approaches (e.g., [8,9,10]). This is generally infeasible if the dimensionality is large. For two subsystems, each with, say, 1000 components, they end up with 1 million causal relations, making it impossible to analyze, albeit with all the details. In this case, the details are not a benefit; they need to be re-analyzed for a big, interpretable picture of the phenomena. On the other hand, in many situations, this is not necessary; one needs only a “bulk” description of the subsystems and their interactions. Such examples are seen from the Reynolds equations for turbulence (e.g., [11]) and the thermodynamic description of molecular motions (e.g., [12]). In some fields (e.g., climate science, neuroscience, geography, etc.), a common practice is simply to take respective averages and form the mean properties, and to study the interactions between the proxies, i.e., the mean properties. A more sophisticated approach is to extract the respective principal components (PCs) (e.g., [13,14,15]), based on which the interactions are analyzed henceforth. These approaches, as we will be examining in this study, however, may not work satisfactorily; their validities need to be carefully checked before being put into application.

During the past 16 years, it has been gradually realized that causality in terms of information flow (IF) is a real physical notion that can be rigorously derived from first principles (see [16]). When two processes interact, IF provides not only the direction but also the strength of the interaction. Thus far, the formalism of the IF between two components has been well established (see [16,17,18,19,20], among others). It has been shown promising to extend the formalism to subspaces with many components involved. A pioneering effort is [21], where the authors show that the heuristic argument in [17] equally applies to that between subsystems in the case with only one-way causality. A recent study on the role of individual nodes in a complex network [22] may be viewed as another effort. (Causality analyses between subspaces with the classical approaches are rare; a few examples are [23,24], etc.) However, a rigorous formalism for more generic problems (e.g., with mutual causality involved) is yet to be implemented. This makes the objective of this study, i.e., to study the interactions between two complex subsystems within a large parental system by investigating the “bulk” information flow between them.

The rest of the paper is organized as follows. In Section 2, we first present the setting for the problem and then derive the IF formulas. Maximum likelihood estimators of these formulas are given in Section 3, which is followed by a validation. Finally, Section 5 summarizes the study.

## 2. Information Flow between Two Subspaces of a Complex System

Consider an *n*-dimensional dynamical system

(1) $$\begin{array}{ccc} & & A:\;\left\{\begin{array}{c} \frac{dx_{1}}{dt}=F_{1}\left(x_{1},x_{2},\ldots ,x_{n};t\right)+\sum_{k=1}^{m}b_{1,k}\left(x_{1},x_{2},\ldots ,x_{n};t\right){\dot{w}}_{k}\\ \;\vdots \;\;\;\;\;\;\;\;\vdots \\ \frac{dx_{r}}{dt}=F_{r}\left(x_{1},x_{2},\ldots ,x_{n};t\right)+\sum_{k=1}^{m}b_{r,k}\left(x_{1},x_{2},\ldots ,x_{n};t\right){\dot{w}}_{k} \end{array}\right. \end{array}$$

(2) $$\begin{array}{ccc} & & B:\;\left\{\begin{array}{c} \frac{dx_{r+1}}{dt}=F_{r+1}\left(x_{1},x_{2},\ldots ,x_{n};t\right)+\sum_{k=1}^{m}b_{r+1,k}\left(x_{1},x_{2},\ldots ,x_{n};t\right){\dot{w}}_{k}\\ \;\vdots \;\;\;\;\;\;\;\;\vdots \\ \frac{dx_{s}}{dt}=F_{s}\left(x_{1},x_{2},\ldots ,x_{n};t\right)+\sum_{k=1}^{m}b_{s,k}\left(x_{1},x_{2},\ldots ,x_{n};t\right){\dot{w}}_{k} \end{array}\right. \end{array}$$

(3) $$\begin{array}{ccc} & & \;\left\{\begin{array}{c} \frac{dx_{s+1}}{dt}=F_{s+1}\left(x_{1},x_{2},\ldots ,x_{n};t\right)+\sum_{k=1}^{m}b_{s+1,k}\left(x_{1},x_{2},\ldots ,x_{n};t\right){\dot{w}}_{k}\\ \;\vdots \;\;\;\;\;\;\;\;\vdots \\ \frac{dx_{n}}{dt}=F_{n}\left(x_{1},x_{2},\ldots ,x_{n};t\right)+\sum_{k=1}^{m}b_{nk}\left(x_{1},x_{2},\ldots ,x_{n};t\right){\dot{w}}_{k}. \end{array}\right. \end{array}$$

where $x\in {\mathbb{R}}^{n}$ denotes the vector of state variable $\left(x_{1},x_{2},\ldots ,x_{n}\right)$, $F=(F_{1},\ldots ,F_{n})$ are differentiable functions of x and time *t*, w is a vector of *m* independent standard Wiener processes, and $B=(b_{ij})$ is an n×m matrix of stochastic perturbation amplitudes. Here we follow the convention in physics not to distinguish a random variable from its deterministic counterpart. From the components $\left(x_{1},\ldots ,x_{n}\right)$, we separate out two sets, $\left(x_{1},\ldots ,x_{r}\right)$ and $\left(x_{r+1},\ldots ,x_{s}\right)$, and denote them as $x_{1\ldots r}$ and $x_{r+1,\ldots ,s}$, respectively. The remaining components $\left(x_{s+1},\ldots ,x_{n}\right)$ are denoted as $x_{s+1,\ldots ,n}$. The subsystems formed by them are henceforth referred to as *A* and *B*, and the following is a derivation of the information flow between them. Note that, for convenience, here *A* and *B* are put adjacent to each other; if not, the equations can always be rearranged to make them so.

Associated with Equations (1)–(3) there is a Fokker–Planck equation governing the evolution of the joint probability density function (pdf) ρ of x:

(4) $$\begin{array}{c} \frac{\partial \rho }{\partial t}+\frac{\partial \rho F_{1}}{\partial x_{1}}+\frac{\partial \rho F_{2}}{\partial x_{2}}+\ldots +\frac{\partial \rho F_{n}}{\partial x_{n}}=\frac{1}{2}\sum_{i=1}^{n}\sum_{j=1}^{n}\frac{\partial^{2}g_{ij}\rho }{\partial x_{i}\partial x_{j}}, \end{array}$$

where $g_{ij}=\sum_{k=1}^{m}b_{ik}b_{jk}$, i,j=1,…,n. Without much loss of generality, ρ is assumed to be compactly supported on ${\mathbb{R}}^{n}$. The joint pdfs of $x_{1\ldots r}$ and $x_{r+1,\ldots ,s}$ are, respectively,

$$\begin{array}{ccc} & & \rho_{1\ldots r}=\int_{{\mathbb{R}}^{n-r}}\rho \left(x\right)dx_{r+1}\ldots dx_{n}\equiv \int_{{\mathbb{R}}^{n-r}}\rho \left(x\right)dx_{r+1,\ldots ,n},\\ & & \rho_{r+1,\ldots ,s}=\int_{{\mathbb{R}}^{n-s+r}}\rho \left(x\right)dx_{1}\ldots dx_{r}dx_{s+1}\ldots dx_{n}\equiv \int_{{\mathbb{R}}^{n-s+r}}\rho \left(x\right)dx_{1,\ldots ,r,s+1,\ldots ,n}. \end{array}$$

With respect to them, the joint entropies are then

(5) $$\begin{array}{ccc} & & H_{A}=-\int_{{\mathbb{R}}^{r}}\rho_{1\ldots r}\log\rho_{1\ldots r}dx_{1\ldots r}, \end{array}$$

(6) $$\begin{array}{ccc} & & H_{B}=-\int_{{\mathbb{R}}^{s-r}}\rho_{r+1,\ldots ,s}\log\rho_{r+1,\ldots ,s}dx_{r+1,\ldots ,s}. \end{array}$$

To derive the evolution of $\rho_{1\ldots r}$, integrate out $\left(x_{r+1},\ldots ,x_{n}\right)$ in Equation (4). This yields, by using the assumption of compactness for ρ,

(7) $$\begin{array}{ccc} & & \frac{\partial \rho_{1\ldots r}}{\partial t}+\sum_{i=1}^{r}\frac{\partial \;}{\partial x_{i}}\int_{{\mathbb{R}}^{n-r}}\rho F_{i}dx_{r+1,\ldots ,n}=\frac{1}{2}\sum_{i=1}^{r}\sum_{j=1}^{r}\int_{{\mathbb{R}}^{n-r}}\frac{\partial^{2}g_{ij}\rho }{\partial x_{i}\partial x_{j}}dx_{r+1,\ldots ,n}. \end{array}$$

Similarly,

(8) $$\begin{array}{ccc} & & \frac{\partial \rho_{r+1,\ldots ,s}}{\partial t}+\sum_{i=r+1}^{s}\frac{\partial \;}{\partial x_{i}}\int_{{\mathbb{R}}^{n-s+r}}\rho F_{i}dx_{1,\ldots ,r,s+1,\ldots ,n}=\frac{1}{2}\sum_{i=r+1}^{s}\sum_{j=r+1}^{s}\int_{{\mathbb{R}}^{n-s+r}}\frac{\partial^{2}g_{ij}\rho }{\partial x_{i}\partial x_{j}}dx_{1,\ldots ,r,s+1,\ldots ,n}. \end{array}$$

Multiplication of Equation (7) by $-(1+\log\rho_{1\ldots r})$, followed by an integration with respect to $x_{1\ldots r}$ over ${\mathbb{R}}^{r}$, yields

$$\begin{array}{ccc} & & \frac{dH_{A}}{dt}-\sum_{i=1}^{r}\int_{{\mathbb{R}}^{r}}\left[\left(1+\log\rho_{1\ldots r}\right)\cdot \frac{\partial \;}{\partial x_{i}}\int_{{\mathbb{R}}^{n-r}}\rho F_{i}dx_{r+1,\ldots ,n}\right]\;dx_{1\ldots r}\\ & & \;=-\frac{1}{2}\int_{{\mathbb{R}}^{r}}\left[\left(1+\log\rho_{1\ldots r}\right)\cdot \sum_{i=1}^{r}\sum_{j=1}^{r}\int_{{\mathbb{R}}^{n-r}}\frac{\partial^{2}g_{ij}\rho }{\partial x_{i}\partial x_{j}}dx_{r+1,\ldots ,n}\right]\;dx_{1\ldots r}. \end{array}$$

Note that in the second term of the left hand side, the part within the summation is, by integration by parts,

$$\begin{array}{ccc} & & \int_{{\mathbb{R}}^{r}}\left(\log\rho_{1\ldots r}\right)\cdot \frac{\partial \;}{\partial x_{i}}\left(\int_{{\mathbb{R}}^{n-r}}\rho F_{i}dx_{r+1,\ldots ,n}\right)dx_{1\ldots r}\\ & & \;=-\int_{{\mathbb{R}}^{r}}\int_{{\mathbb{R}}^{n-r}}\rho F_{i}\frac{\partial \log\rho_{1\ldots r}}{\partial x_{i}}dx_{r+1,\ldots ,n}dx_{1\ldots r}\\ & & \;=-\int_{{\mathbb{R}}^{n}}\rho F_{i}\frac{\partial \log\rho_{1\ldots r}}{\partial x_{i}}\;dx\\ & & \;=-E\left[F_{i}\frac{\partial \log\rho_{1\ldots r}}{\partial x_{i}}\right]. \end{array}$$

In the derivation, the compactness assumption has been used (variables vanish at the boundaries). By the same approach, the right hand side becomes

$$\begin{array}{ccc} & & -\frac{1}{2}\int_{{\mathbb{R}}^{r}}\left[\log\rho_{1\ldots r}\cdot \sum_{i=1}^{r}\sum_{j=1}^{r}\int_{{\mathbb{R}}^{n-r}}\frac{\partial^{2}g_{ij}\rho }{\partial x_{i}\partial x_{j}}dx_{r+1,\ldots ,n}\right]dx_{1\ldots r}\\ & & \;=-\frac{1}{2}\sum_{i=1}^{r}\sum_{j=1}^{r}\int_{{\mathbb{R}}^{n}}\left[\log\rho_{1\ldots r}\cdot \frac{\partial^{2}g_{ij}\rho }{\partial x_{i}\partial x_{j}}\right]dx\\ & & \;=-\frac{1}{2}\sum_{i=1}^{r}\sum_{j=1}^{r}\int_{{\mathbb{R}}^{n}}\left[g_{ij}\rho \frac{\partial^{2}\log\rho_{1\ldots r}}{\partial x_{i}\partial x_{j}}\right]dx\\ & & \;=-\frac{1}{2}\sum_{i=1}^{r}\sum_{j=1}^{r}E\left[g_{ij}\frac{\partial^{2}\log\rho_{1\ldots r}}{\partial x_{i}\partial x_{j}}\right]. \end{array}$$

Hence,

(9) $$\begin{array}{c} \frac{dH_{A}}{dt}=-\sum_{i=1}^{r}E\left[F_{i}\frac{\partial \log\rho_{1\ldots r}}{\partial x_{i}}\right]-\frac{1}{2}\sum_{i=1}^{r}\sum_{j=1}^{r}E\left[g_{ij}\frac{\partial^{2}\log\rho_{1\ldots r}}{\partial x_{i}\partial x_{j}}\right]. \end{array}$$

Likewise, we have

(10) $$\begin{array}{c} \frac{dH_{B}}{dt}=-\sum_{i=r+1}^{s}E\left[F_{i}\frac{\partial \log\rho_{r+1,\ldots ,s}}{\partial x_{i}}\right]-\frac{1}{2}\sum_{i=r+1}^{s}\sum_{j=r+1}^{s}E\left[g_{ij}\frac{\partial^{2}\log\rho_{r+1,\ldots ,s}}{\partial x_{i}\partial x_{j}}\right]. \end{array}$$

Now consider the impact of the subsystem *A* on its peer *B*, written $T_{A\to B}$. Following Liang (2016) [16], this is associated with the evolution of the joint entropy of the latter:

(11) $$\begin{array}{c} \frac{dH_{B}}{dt}=\frac{dH_{B\setminus \;A}}{dt}+T_{A\to B}, \end{array}$$

where $H_{B\setminus \;A}$ signifies the entropy evolution with the influence of *A* excluded, which is found by instantaneously freezing $\left(x_{1},\ldots ,x_{r}\right)\equiv x_{1\ldots r}$ as parameters. To do this, examine, on an infinitesimal interval [t,t+Δt], a system modified from the original Equations (1)–(3) by removing the *r* equations for $x_{1}$, $x_{2}$, ..., $x_{r}$ from the equation set

(12) $$\begin{array}{ccc} & & \frac{dx_{r+1}}{dt}=F_{r+1}\left(x_{1},x_{2},\ldots ,x_{n};t\right)+\sum_{k=1}^{m}b_{r+1,k}\left(x_{1},x_{2},\ldots ,x_{n};t\right){\dot{w}}_{k} \end{array}$$

$$\begin{array}{c} \;\;\;\;\;\;\vdots \;\;\;\;\;\;\;\;\;\vdots \end{array}$$

(13) $$\begin{array}{ccc} & & \frac{dx_{s}}{dt}=F_{s}\left(x_{1},x_{2},\ldots ,x_{n};t\right)+\sum_{k=1}^{m}b_{s,k}\left(x_{1},x_{2},\ldots ,x_{n};t\right){\dot{w}}_{k} \end{array}$$

(14) $$\begin{array}{ccc} & & \frac{dx_{s+1}}{dt}=F_{s+1}\left(x_{1},x_{2},\ldots ,x_{n};t\right)+\sum_{k=1}^{m}b_{s+1,k}\left(x_{1},x_{2},\ldots ,x_{n};t\right){\dot{w}}_{k} \end{array}$$

$$\begin{array}{c} \;\;\;\;\;\;\vdots \;\;\;\;\;\;\;\;\;\vdots \end{array}$$

(15) $$\begin{array}{ccc} & & \frac{dx_{n}}{dt}=F_{n}\left(x_{1},x_{2},\ldots ,x_{n};t\right)+\sum_{k=1}^{m}b_{nk}\left(x_{1},x_{2},\ldots ,x_{n};t\right){\dot{w}}_{k}. \end{array}$$

Notice that the $F_{i}$s and $b_{ik}$s still have dependence on $\left(x_{1},\ldots ,x_{r}\right)\equiv x_{1\ldots r}$, which, however, appear in the modified system as parameters. By [16], we can construct a mapping $\Phi :{\mathbb{R}}^{n-r}\to {\mathbb{R}}^{n-r}$, $x_{\setminus \;A}\left(t\right)\mapsto x_{\setminus \;A}\left(t+\Delta t\right)$, where $x_{\setminus \;A}$ means x but with $x_{1\ldots r}$ appearing as parameters, and study the Frobenius–Perron operator (see, for example, [25]) of the modified system. An alternative approach is given by Liang in [18], which we henceforth follow. Observe that on the interval [t,t+Δt], corresponding to the modified dynamical system, there is also a Fokker–Planck equation

$$\begin{array}{ccc} & & \frac{\partial \rho_{\setminus \;A}}{\partial t}+\sum_{i=r+1}^{n}\frac{\partial F_{i}\rho_{\setminus \;A}}{\partial x_{i}}=\frac{1}{2}\sum_{i=r+1}^{n}\sum_{j=r+1}^{n}\frac{\partial^{2}g_{ij}\rho_{\setminus \;A}}{\partial x_{i}\partial x_{j}},\\ & & \rho_{\setminus \;A}=\rho_{r+1,\ldots ,n}\;\;\;\mathrm{at}\;\mathrm{time}\;t. \end{array}$$

Here $g_{ij}=\sum_{k=1}^{m}b_{ik}b_{jk}$, $\rho_{\setminus \;A}$ means the joint pdf of $\left(x_{r+1},\ldots ,x_{n}\right)$ with $x_{1\ldots r}$ frozen as parameters. Note the difference between $\rho_{\setminus \;A}$ and $\rho_{r+1,\ldots ,n}$; the former has $x_{1\ldots r}$ as parameters, while the latter has no dependence on $x_{1\ldots r}$. However, they are equal at time *t*.

Integration of the above Fokker–Planck equation with respect to $dx_{s+1,\ldots ,n}$ gives the evolution of the pdf of subsystem *B* with *A* frozen as parameters, written $\rho_{B,\setminus \;A}$:

(16) $$\begin{array}{ccc} & & \frac{\partial \rho_{B,\setminus \;A}}{\partial t}+\sum_{i=r+1}^{s}\int_{{\mathbb{R}}^{n-s}}\frac{\partial F_{i}\rho_{\setminus \;A}}{\partial x_{i}}dx_{s+1,\ldots ,n}=\frac{1}{2}\sum_{i=r+1}^{s}\sum_{j=r+1}^{s}\int_{{\mathbb{R}}^{n-s}}\frac{\partial^{2}g_{ij}\rho_{\setminus \;A}}{\partial x_{i}\partial x_{j}}dx_{s+1,\ldots ,n}, \end{array}$$

(17) $$\begin{array}{ccc} & & \rho_{B,\setminus \;A}=\rho_{r+1,\ldots ,s}\;\;\;\mathrm{at}\;\mathrm{time}\;t. \end{array}$$

Divide Equation (16) by $\rho_{B,\setminus \;A}$ and simplify the notation $x_{r+1,\ldots ,s}$ by $x_{B}$ to obtain

$$\begin{array}{c} \frac{\partial \log\rho_{B,\setminus \;A}}{\partial t}+\sum_{i=r+1}^{s}\frac{1}{\rho_{B,\setminus \;A}}\int_{{\mathbb{R}}^{n-s}}\frac{\partial F_{i}\rho_{\setminus \;A}}{\partial x_{i}}dx_{s+1,\ldots ,n}=\frac{1}{2\rho_{B,\setminus \;A}}\sum_{i=r+1}^{s}\sum_{j=r+1}^{s}\int_{{\mathbb{R}}^{n-s}}\frac{\partial^{2}g_{ij}\rho_{\setminus \;A}}{\partial x_{i}\partial x_{j}}dx_{s+1,\ldots ,n}. \end{array}$$

Discretizing and noticing that $\rho_{B,\setminus \;A}\left(t\right)=\rho_{r+1,\ldots ,s}\left(t\right)$, we have (in the following, unless otherwise indicated, the variables without arguments explicitly specified are assumed to be at time step *t*)

$$\begin{array}{ccc} & & \log\rho_{B,\setminus \;A}\left(x_{B};t+\Delta t\right)\\ & & =\log\rho_{r+1,\ldots ,s}\left(x_{B};t\right)-\Delta t\cdot \sum_{i=r+1}^{s}\frac{1}{\rho_{r+1,\ldots ,s}}\int_{{\mathbb{R}}^{n-s}}\frac{\partial F_{i}\rho_{r+1,\ldots ,n}}{\partial x_{i}}dx_{s+1,\ldots ,n}\\ & & \;+\frac{\Delta t}{2}\sum_{i=r+1}^{s}\sum_{j=r+1}^{s}\frac{1}{\rho_{r+1,\ldots ,s}}\int_{{\mathbb{R}}^{n-s}}\frac{\partial^{2}g_{ij}\rho_{r+1,\ldots ,n}}{\partial x_{i}\partial x_{j}}dx_{s+1,\ldots ,n}+o\left(\Delta t\right). \end{array}$$

To arrive at $dH_{B,\setminus \;A}/dt$, we need to find $\log\rho_{B,\setminus \;A}\left(x_{B}\left(t+\Delta t\right);t+\Delta t\right)$. Using the Euler–Bernstein approximation,

(18) $$\begin{array}{c} x_{B}\left(t+\Delta t\right)=x_{B}\left(t\right)+F_{B}\Delta t+B_{B}\Delta w \end{array}$$

where, just as the notation $x_{B}$,

$$\begin{array}{ccc} & & F_{B}={\left(F_{r+1},\ldots ,F_{s}\right)}^{T},\\ & & B_{B}=\left[\begin{array}{ccc} b_{r+1,1} & \cdots & b_{r+1,m}\\ \vdots & \ddots & \vdots \\ b_{s1} & \cdots & b_{sm} \end{array}\right]\\ & & \Delta w={\left(\Delta w_{1},\ldots ,\Delta w_{m}\right)}^{T} \end{array}$$

and $\Delta w_{k}∼N\left(0,\Delta t\right)$, we have

$$\begin{array}{ccc} & & \log(\rho_{B,\setminus \;A}\left(x_{B}\left(t+\Delta t\right);t+\Delta t\right)\\ & & =\log\rho_{r+1,\ldots ,s}\left(x_{B}\left(t\right)+F_{B}\Delta t+B_{B}\Delta w;t\right)\\ & & \;\;\;-\Delta t\cdot \sum_{r+1}^{s}\frac{1}{\rho_{r+1,\ldots ,s}}\int_{{\mathbb{R}}^{n-s}}\frac{\partial F_{i}\rho_{r+1,\ldots ,n}}{\partial x_{i}}dx_{s+1,\ldots ,n}+\frac{\Delta t}{2}\sum_{r+1}^{s}\sum_{r+1}^{s}\frac{1}{\rho_{r+1,\ldots ,s}}\int_{{\mathbb{R}}^{n-s}}\frac{\partial^{2}g_{ij}\rho_{r+1,\ldots ,n}}{\partial x_{i}\partial x_{j}}dx_{s+1,\ldots ,n}+o\left(\Delta t\right).\\ & & =\log\rho_{r+1,\ldots ,s}\left(x_{B}\left(t\right)\right)+\sum_{i=r+1}^{s}\left[\frac{\partial \log\rho_{r+1,\ldots ,s}}{\partial x_{i}}\left(F_{i}\Delta t+\sum_{k=1}^{m}b_{ik}\Delta w_{k}\right)\right]\\ & & \;\;\;+\frac{1}{2}\cdot \sum_{i=r+1}^{s}\sum_{j=r+1}^{s}\left[\frac{\partial^{2}\log\rho_{r+1,\ldots ,s}}{\partial x_{i}\partial x_{j}}\left(F_{i}\Delta t+\sum_{k=1}^{m}b_{ik}\Delta w_{k}\right)\cdot \left(F_{j}\Delta t+\sum_{l=1}^{m}b_{jl}\Delta w_{l}\right)\right]\\ & & \;\;\;-\Delta t\cdot \sum_{r+1}^{s}\frac{1}{\rho_{r+1,\ldots ,s}}\int_{{\mathbb{R}}^{n-s}}\frac{\partial F_{i}\rho_{r+1,\ldots ,n}}{\partial x_{i}}dx_{s+1,\ldots ,n}+\frac{\Delta t}{2}\sum_{r+1}^{s}\sum_{r+1}^{s}\frac{1}{\rho_{r+1,\ldots ,s}}\int_{{\mathbb{R}}^{n-s}}\frac{\partial^{2}g_{ij}\rho_{r+1,\ldots ,n}}{\partial x_{i}\partial x_{j}}dx_{s+1,\ldots ,n}+o\left(\Delta t\right). \end{array}$$

Take mathematical expectation on both sides. The left hand side is $-H_{B,\setminus \;A}\left(t+\Delta t\right)$. By Corollary III.I of [16], and noting $E\Delta w_{k}=0$, $E\Delta w_{k}^{2}=\Delta t$ and the fact that Δw are independent of $x_{B}$, we have

$$\begin{array}{ccc} & & -H_{B,\setminus \;A}\left(t+\Delta t\right)=-H_{B}\left(t\right)+\Delta t\cdot E\sum_{i=r+1}^{s}F_{i}\frac{\partial \log\rho_{r+1,\ldots ,s}}{\partial x_{i}}\\ & & \;+\frac{\Delta t}{2}\cdot E\sum_{i=r+1}^{s}\sum_{j=r+1}^{s}\sum_{k=1}^{m}\sum_{l=1}^{m}b_{ik}b_{jl}\delta_{kl}\frac{\partial^{2}\log\rho_{r+1,\ldots ,s}}{\partial x_{i}\partial x_{j}}\\ & & \;-\Delta t\cdot E\sum_{r+1}^{s}\frac{1}{\rho_{r+1,\ldots ,s}}\int_{{\mathbb{R}}^{n-s}}\frac{\partial F_{i}\rho_{r+1,\ldots ,n}}{\partial x_{i}}dx_{s+1,\ldots ,n}\\ & & \;+\frac{\Delta t}{2}E\sum_{r+1}^{s}\sum_{r+1}^{s}\frac{1}{\rho_{r+1,\ldots ,s}}\int_{{\mathbb{R}}^{n-s}}\frac{\partial^{2}g_{ij}\rho_{r+1,\ldots ,n}}{\partial x_{i}\partial x_{j}}dx_{s+1,\ldots ,n}+o\left(\Delta t\right)\\ & & =-H_{B}\left(t\right)+\Delta t\cdot E\sum_{i=r+1}^{s}F_{i}\frac{\partial \log\rho_{r+1,\ldots ,s}}{\partial x_{i}}+\frac{\Delta t}{2}\cdot E\sum_{i=r+1}^{s}\sum_{j=r+1}^{s}g_{ij}\frac{\partial^{2}\log\rho_{r+1,\ldots ,s}}{\partial x_{i}\partial x_{j}}\\ & & \;-\Delta t\cdot E\sum_{r+1}^{s}\frac{1}{\rho_{r+1,\ldots ,s}}\int_{{\mathbb{R}}^{n-s}}\frac{\partial F_{i}\rho_{r+1,\ldots ,n}}{\partial x_{i}}dx_{s+1,\ldots ,n}\\ & & \;+\frac{\Delta t}{2}E\sum_{r+1}^{s}\sum_{r+1}^{s}\frac{1}{\rho_{r+1,\ldots ,s}}\int_{{\mathbb{R}}^{n-s}}\frac{\partial^{2}g_{ij}\rho_{r+1,\ldots ,n}}{\partial x_{i}\partial x_{j}}dx_{s+1,\ldots ,n}+o\left(\Delta t\right). \end{array}$$

Thus,

$$\begin{array}{ccc} & & \frac{dH_{B,\setminus \;A}}{dt}=\lim_{\Delta t\to 0}\frac{H_{B,\setminus \;A}-H_{B}\left(t\right)}{\Delta t}\\ & & \;=-E\sum_{i=r+1}^{n}\left(F_{i}\frac{\partial \log\rho_{r+1,\ldots ,s}}{\partial x_{i}}-\frac{1}{\rho_{r+1,\ldots ,s}}\int_{{\mathbb{R}}^{n-s}}\frac{\partial F_{i}\rho_{r+1,\ldots ,n}}{\partial x_{i}}dx_{s+1,\ldots ,n}\right)\\ & & \;\;-\frac{1}{2}E\sum_{i=r+1}^{s}\sum_{j=r+1}^{s}\left(g_{ij}\frac{\partial^{2}\log\rho_{r+1,\ldots ,s}}{\partial x_{i}\partial x_{j}}+\frac{1}{\rho_{r+1,\ldots ,s}}\frac{\partial^{2}\;}{\partial x_{i}\partial x_{j}}\int_{{\mathbb{R}}^{n-s}}g_{ij}\rho_{r+1,\ldots ,n}dx_{s+1,\ldots ,n}\right). \end{array}$$

Hence, the information flow from $x_{1\ldots r}$ to $x_{r+1,\ldots ,s}$ is

$$\begin{array}{ccc} & & T_{A\to B}=\frac{dH_{B}}{dt}-\frac{dH_{B,\setminus \;A}}{dt}\\ & & \;=-E\sum_{i=r+1}^{s}\left(F_{i}\frac{\partial \log\rho_{r+1,\ldots ,s}}{\partial x_{i}}\right)-\frac{1}{2}E\sum_{i=r+1}^{s}\sum_{j=r+1}^{s}\left(g_{ij}\frac{\partial^{2}\log\rho_{r+1,\ldots ,s}}{\partial x_{i}\partial x_{j}}\right)\\ & & \;\;\;\;\;+E\sum_{i=r+1}^{s}\left(F_{i}\frac{\partial \log\rho_{r+1,\ldots ,s}}{\partial x_{i}}-\frac{1}{\rho_{r+1,\ldots ,s}}\int_{{\mathbb{R}}^{n-s}}\frac{\partial F_{i}\rho_{r+1,\ldots ,n}}{\partial x_{i}}dx_{s+1,\ldots ,n}\right)\\ & & \;\;\;\;\;+\frac{1}{2}E\sum_{i=r+1}^{s}\sum_{j=r+1}^{s}\left(g_{ij}\frac{\partial^{2}\log\rho_{r+1,\ldots ,s}}{\partial x_{i}\partial x_{j}}+\frac{1}{\rho_{r+1,\ldots ,s}}\int_{{\mathbb{R}}^{n-s}}\frac{\partial^{2}g_{ij}\rho_{r+1,\ldots ,n}}{\partial x_{i}\partial x_{j}}dx_{s+1,\ldots ,n}\right)\\ & & \;=-E\left[\sum_{i=r+1}^{s}\frac{1}{\rho_{r+1,\ldots ,s}}\int_{{\mathbb{R}}^{n-s}}\frac{\partial F_{i}\rho_{r+1,\ldots ,n}}{\partial x_{i}}dx_{s+1,\ldots ,n}\right]\\ & & \;\;\;\;\;+\frac{1}{2}E\left[\sum_{i=r+1}^{s}\sum_{j=r+1}^{s}\frac{1}{\rho_{r+1,\ldots ,s}}\int_{{\mathbb{R}}^{n-s}}\frac{\partial^{2}g_{ij}\rho_{r+1,\ldots ,n}}{\partial x_{i}\partial x_{j}}dx_{s+1,\ldots ,n}\right]. \end{array}$$

Likewise, we can obtain the information flow from subsystem *B* to subsystem *A*. These are summarized in the following theorem.

**Theorem** **1.** *For the dynamical system Equations (1)–(3), if the probability density function (pdf) of x is compactly supported, then the information flow from $x_{1\ldots r}$ to $x_{r+1,\ldots ,s}$ and that from $x_{r+1,\ldots ,s}$ to $x_{1\ldots r}$ are (in nats per unit time), respectively,*

(19) $$\begin{array}{ccc} & & T_{A\to B}=-E\left[\sum_{i=r+1}^{s}\frac{1}{\rho_{r+1,\ldots ,s}}\int_{{\mathbb{R}}^{n-s}}\frac{\partial F_{i}\rho_{r+1,\ldots ,n}}{\partial x_{i}}dx_{s+1,\ldots ,n}\right]\\ & & \;\;\;\;\;+\frac{1}{2}E\left[\sum_{i=r+1}^{s}\sum_{j=r+1}^{s}\frac{1}{\rho_{r+1,\ldots ,s}}\int_{{\mathbb{R}}^{n-s}}\frac{\partial^{2}g_{ij}\rho_{r+1,\ldots ,n}}{\partial x_{i}\partial x_{j}}dx_{s+1,\ldots ,n}\right], \end{array}$$

(20) $$\begin{array}{ccc} & & T_{B\to A}=-E\left[\sum_{i=1}^{r}\frac{1}{\rho_{1\ldots r}}\int_{{\mathbb{R}}^{n-s}}\frac{\partial F_{i}\rho_{1,\ldots ,r,s+1,\ldots ,n}}{\partial x_{i}}dx_{s+1,\ldots ,n}\right]\\ & & \;\;\;\;\;+\frac{1}{2}E\left[\sum_{i=1}^{r}\sum_{j=1}^{r}\frac{1}{\rho_{1\ldots r}}\int_{{\mathbb{R}}^{n-s}}\frac{\partial^{2}g_{ij}\rho_{1,\ldots ,r,s+1,\ldots ,n}}{\partial x_{i}\partial x_{j}}dx_{s+1,\ldots ,n}\right], \end{array}$$

*where $g_{ij}=\sum_{k=1}^{m}b_{ik}b_{jk}$, and E signifies mathematical expectation.*

When r=1, s=n=2, (20) reduces to

$$\begin{array}{c} T_{B\to A}=-E\left[\frac{1}{\rho_{1}}\frac{\partial F_{1}\rho_{1}}{\partial x_{1}}\right]+\frac{1}{2}E\left[\frac{1}{\rho_{1}}\frac{\partial^{2}g_{11}\rho_{1}}{\partial x_{1}^{2}}\right] \end{array}$$

which is precisely the same as the Equation (15) in [18]; the same holds for Equation (19). These equations are hence verified.

The following theorem forms the basis for causal inference.

**Theorem** **2.** *If the evolution of subsystem A (resp. B) does not depend on $x_{r+1,\ldots ,s}$ (resp. $x_{1\ldots r}$), then $T_{B\to A}=0$ (resp. $T_{A\to B}=0$).*

**Proof.**  We only check the formula for $T_{B\to A}$. In (20), the deterministic part

$$\begin{array}{ccc} & & -E\left[\sum_{i=1}^{r}\frac{1}{\rho_{1\ldots r}}\int_{{\mathbb{R}}^{n-s}}\frac{\partial F_{i}\rho_{1,\ldots ,r,s+1,\ldots ,n}}{\partial x_{i}}dx_{s+1,\ldots ,n}\right]\\ & & =-\sum_{i=1}^{r}\int_{{\mathbb{R}}^{r}}\int_{{\mathbb{R}}^{s-r}}\left[\left(\frac{\rho_{1,\ldots ,s}}{\rho_{1\ldots r}}\right)\int_{{\mathbb{R}}^{n-s}}\frac{\partial F_{i}\rho_{1,\ldots ,r,s+1,\ldots ,n}}{\partial x_{i}}dx_{s+1,\ldots ,n}\right]dx_{1\ldots r}dx_{r+1,\ldots ,s}. \end{array}$$

Now, $F_{i}$ is independent of $x_{r+1,\ldots ,s}$, and note that $\rho_{1,\ldots ,r,s+1,\ldots ,n}$ is also so. Thus, we may integrate $\rho_{1,\ldots ,s}$ within the parentheses directly with respect to $dx_{r+1,\ldots ,s}$, yielding

$$\begin{array}{c} \frac{\int_{{\mathbb{R}}^{s-r}}\rho_{1,\ldots ,s}dx_{r+1,\ldots ,s}}{\rho_{1\ldots r}}=\frac{\rho_{1\ldots r}}{\rho_{1\ldots r}}=1. \end{array}$$

By the compactness of ρ, the whole deterministic part hence vanishes. Likewise, it can be proved that the stochastic part also vanishes.

This theorem allows us to identify the causality with information flow. Ideally, if $T_{B\to A}=0$, then *B* is not causal to *A*, and vice versa; the same holds for $T_{A\to B}$. □

## 3. Information Flow between Linear Subsystems and Its Estimation

Linear systems provide the simplest framework which is usually taken as the first step toward a more generic setting. Simple as it may be, it has been demonstrated in practice that linear results often provide a good approximation of an otherwise much more complicated problem. It is hence of interest to examine this special case.

Let

(21) $$\begin{array}{c} F_{i}=f_{i}+\sum_{j=1}^{n}a_{ij}x_{j}, \end{array}$$

where $f_{i}$ and $a_{ij}$ are constants. Additionally, suppose that $b_{ij}$ are constants—that is to say, the noises are additive. Then, $g_{ij}$ are also constants. Thus, in Equation (20),

$$\begin{array}{ccc} & & E\left(\frac{1}{\rho_{1\ldots r}}\int_{n-s}\frac{\partial^{2}g_{ij}\rho_{1,\ldots ,r,s+1,\ldots ,n}}{\partial x_{i}\partial x_{j}}dx_{s+1,\ldots ,n}\right)\\ & & \;=g_{ij}\int_{{\mathbb{R}}^{s}}\rho \left(x_{1\ldots s}\right)\frac{1}{\rho_{1\ldots r}}\frac{\partial^{2}\int_{}\rho_{1,\ldots ,r,s+1,\ldots ,n}}{\partial x_{i}\partial x_{j}}dx_{s+1,\ldots ,n}dx_{1\ldots s}\\ & & \;=g_{ij}\int_{{\mathbb{R}}^{r}}\int_{{\mathbb{R}}^{s-r}}\frac{\rho_{1\ldots s}}{\rho_{1\ldots r}}\frac{\partial^{2}\rho_{1\ldots r}}{\partial x_{i}\partial x_{j}}dx_{r+1,\ldots ,s}dx_{1\ldots r}\\ & & \;=g_{ij}\int_{{\mathbb{R}}^{r}}1\cdot \frac{\partial^{2}\rho_{1\ldots r}}{\partial x_{i}\partial x_{j}}dx_{1\ldots r}\\ & & \;=0. \end{array}$$

The same holds in Equation (19). Thus, the stochastic parts in both Equations (19) and (20) vanish.

Since a linear system initialized with a Gaussian process will always be Gaussian, we may write the joint pdf of x as

(22) $$\begin{array}{c} \rho \left(x_{1},\ldots ,x_{n}\right)=\frac{1}{\sqrt{{\left(2\pi \right)}^{n}\det\Sigma }}e^{-\frac{1}{2}{\left(x-\mu \right)}^{T}\Sigma^{-1}\left(x-\mu \right)}, \end{array}$$

where $\Sigma ={\left(\sigma_{ij}\right)}_{n\times n}$ is the population covariance matrix of x. By the property of the Gaussian process, it is easy to show

(23) $$\begin{array}{c} \rho_{r+1,\ldots ,s}\left(x_{r+1},\ldots ,x_{s}\right)=\frac{1}{\sqrt{{\left(2\pi \right)}^{s-r}\det\Sigma_{B}}}e^{-\frac{1}{2}{\left(x_{B}-\mu_{B}\right)}^{T}\Sigma_{B}^{-1}\left(x_{B}-\mu_{B}\right)}, \end{array}$$

where $x_{B}=\left(x_{r+1},\ldots ,x_{s}\right)$, $\mu_{B}=\left(\mu_{r+1},\ldots ,\mu_{s}\right)$ is the vector of the means of $x_{B}$, and $\Sigma_{B}$ the covariance matrix of $x_{B}$. For easy correspondence, we will augment $x_{B}$, $\mu_{B}$, and $\Sigma_{B}$, so that their entries have the same indices as their counterparts in x, μ and Σ. Separate $F_{i}$ into two parts:

$$\begin{array}{ccc} F_{i} & = & \left[f_{i}+\sum_{j=1}^{r}a_{ij}x_{j}+\sum_{j=s+1}^{n}a_{ij}x_{j}\right]+\left[\sum_{j=r+1}^{s}a_{ij}x_{j}\right]\\ & \equiv & F_{i}^{\prime }+F_{i}^{\prime }, \end{array}$$

where $F_{i}^{\prime }$ and $F_{i}^{\prime }$ correspond to the respective parts in the two square brackets. Thus, $F_{i}^{\prime }$ has nothing to do with the subspace *B*. By Theorem 2, this part does not contribute to the causality from *A* to *B*, so we only need to consider $F_{i}^{\prime \prime }$ in evaluating $T_{A\to B}$; that is to say,

$$\begin{array}{ccc} T_{A\to B} & = & -E\left[\sum_{i=r+1}^{s}\frac{1}{\rho_{r+1,\ldots ,s}}\frac{\partial \;}{\partial x_{i}}\int_{{\mathbb{R}}^{n-s}}F_{i}\rho_{r+1,\ldots ,n}dx_{s+1,\ldots ,n}\right]\\ & = & -E\left[\sum_{i=r+1}^{s}\frac{1}{\rho_{r+1,\ldots ,s}}\frac{\partial \;}{\partial x_{i}}\int_{{\mathbb{R}}^{n-s}}F_{i}^{\prime \prime }\rho_{r+1,\ldots ,n}dx_{s+1,\ldots ,n}\right]\\ & = & -E\left[\sum_{i=r+1}^{s}\frac{1}{\rho_{r+1,\ldots ,s}}\frac{\partial F_{i}^{\prime \prime }\rho_{r+1,\ldots ,s}}{\partial x_{i}}\right]\\ & = & -\sum_{i=r+1}^{s}\left[E\left(F_{i}^{\prime \prime }\frac{\partial \log\rho_{r+1,\ldots ,s}}{\partial x_{i}}\right)+E\left(\frac{\partial F_{i}^{\prime \prime }}{\partial x_{i}}\right)\right]. \end{array}$$

The second term in the bracket is $a_{ii}$. The first term is

$$\begin{array}{ccc} & & F_{i}^{\prime \prime }\frac{\partial \log\rho_{r+1,\ldots ,s}}{\partial x_{i}}=\left[\sum_{j=r+1}^{s}a_{ij}x_{j}\right]\cdot \frac{\partial \;}{\partial x_{i}}\left[-\frac{1}{2}{\left(x_{B}-\mu_{B}\right)}^{T}\Sigma_{B}^{-1}\left(x_{B}-\mu_{B}\right)\right]\\ & & =\left(\sum_{j=r+1}^{s}a_{ij}x_{j}\right){\dot{\sum }}_{j=r+1}^{s}\left(-\frac{\sigma_{ij}^{\prime }+\sigma_{ji}^{\prime }}{2}\right)\cdot \left(x_{j}-\mu_{j}\right). \end{array}$$

Here, $\sigma_{ij}^{\prime }$ is the (i,j)th entry of the matrix

$$\begin{array}{c} \left[\begin{array}{ccc} I & 0 & 0\\ 0 & \Sigma_{B}^{-1} & 0\\ 0 & 0 & I \end{array}\right]. \end{array}$$

Since, here, only 1≤i,j≤s are in question, this is equal to the (i,j)th entry of the matrix

$$\begin{array}{c} \left[\begin{array}{cc} I_{r\times r} & 0_{r\times (s-r)}\\ 0_{(s-r)\times r} & \Sigma_{B}^{-1} \end{array}\right] \end{array}$$

As $\Sigma_{B}$ is symmetric, so is $\Sigma_{B}^{-1}$, and hence $\left(\sigma_{ij}^{\prime }+\sigma_{ji}^{\prime }\right)/2=\sigma_{ij}^{\prime }$. Thus,

$$\begin{array}{ccc} & & -EF_{i}\frac{\partial \log\rho_{r+1,\ldots ,s}}{\partial x_{i}}=-E\sum_{j=1}^{s}a_{ij}x_{j}\cdot \sum_{j=r+1}^{s}\left(-\sigma_{ij}^{\prime }\right)\cdot \left(x_{j}-\mu_{j}\right)\\ & & =E\sum_{k=1}^{s}a_{ik}\left(x_{k}-\mu_{k}\right)\cdot \sum_{j=r+1}^{s}\sigma_{ij}^{\prime }\left(x_{j}-\mu_{j}\right)\\ & & =\sum_{k=1}^{s}\sum_{j=r+1}^{s}a_{ik}\sigma_{ij}^{\prime }E\left(x_{k}-\mu_{k}\right)\left(x_{j}-\mu_{j}\right)\\ & & =\sum_{k=1}^{s}\sum_{j=r+1}^{s}a_{ik}\sigma_{ij}^{\prime }\sigma_{k}j. \end{array}$$

Substituting back, we obtain a very simplified result for $T_{A\to B}$. Likewise, $T_{B\to A}$ can also be obtained, as shown in the following.

**Theorem** **3.** *In Equations (1)–(3), suppose $b_{ij}$ are constants, and*

(24) $$\begin{array}{ccc} & & F_{i}=f_{i}+\sum_{j=1}^{n}a_{ij}x_{j}, \end{array}$$

*where $f_{i}$ and $a_{ij}$ are also constants. Furthermore, suppose that initially x has a Gaussian distribution; then,*

(25) $$\begin{array}{ccc} T_{A\to B} & = & \sum_{i=r+1}^{s}\left[\sum_{j=r+1}^{s}\sigma_{ij}^{\prime }\left(\sum_{k=1}^{s}a_{ik}\sigma_{kj}\right)-a_{ii}\right], \end{array}$$

*where $\sigma_{ij}^{\prime }$ is the (i,j)th entry of $\left[\begin{array}{cc} I_{r\times r} & 0\\ 0 & \Sigma_{B}^{-1} \end{array}\right]$, and*

(26) $$\begin{array}{ccc} T_{B\to A} & = & \sum_{i=1}^{r}\left[\sum_{j=1}^{r}\sigma_{ij}^{\prime \prime }\left(\sum_{k=1}^{s}a_{ik}\sigma_{kj}\right)-a_{ii}\right], \end{array}$$

*where $\sigma_{ij}^{\prime \prime }$ is the (i,j)th entry of $\left[\begin{array}{cc} \Sigma_{A}^{-1} & 0\\ 0 & I_{\left(s-r)\times (s-r\right)} \end{array}\right]$.*

Given a system such as (1)–(3), we can evaluate in a precise sense the information flows among the components. Now, suppose that instead of the dynamical system, what we have are just *n* time series with *K* steps, K≫n, $\left\{x_{1}\left(k\right)\right\},\left\{x_{2}\left(k\right)\right\},\ldots ,\left\{x_{n}\left(k\right)\right\}$. We can estimate the system from the series and then apply the information flow formula to fulfill the task. Assume a linear model as shown above, and assume m=1. following Liang (2014) [19], the maximum likelihood estimator (mle) of $a_{ij}$ is equal to the least-square solution of the following over-determined problem:

$$\begin{array}{c} \left(\begin{array}{ccccc} 1 & x_{1}\left(1\right) & x_{2}\left(1\right) & \ldots & x_{n}\left(1\right)\\ 1 & x_{1}\left(2\right) & x_{2}\left(2\right) & \ldots & x_{n}\left(2\right)\\ 1 & x_{1}\left(3\right) & x_{2}\left(3\right) & \ldots & x_{n}\left(3\right)\\ \vdots & \vdots & \vdots \ddots & \vdots \\ 1 & x_{1}\left(K\right) & x_{2}\left(K\right) & \ldots & x_{n}\left(K\right) \end{array}\right)\left(\begin{array}{c} f_{i}\\ a_{i1}\\ a_{i2}\\ \vdots \\ a_{in} \end{array}\right)=\left(\begin{array}{c} {\dot{x}}_{i}\left(1\right)\\ {\dot{x}}_{i}\left(2\right)\\ {\dot{x}}_{i}\left(3\right)\\ \vdots \\ {\dot{x}}_{i}\left(K\right) \end{array}\right) \end{array}$$

where ${\dot{x}}_{i}\left(k\right)=\left(x_{i}\left(k+1\right)-x_{i}\left(k\right)\right)/\Delta t$ (Δt is the time stepsize), for i=1,2,…,n, k=1,…,K. Use an overbar to denote the time mean over the *K* steps. The above equation is

$$\begin{array}{c} \left(\begin{array}{ccccc} 1 & {\bar{x}}_{1} & {\bar{x}}_{2} & \ldots & {\bar{x}}_{n}\\ 0 & x_{1}\left(2\right)-{\bar{x}}_{1} & x_{2}\left(2\right)-{\bar{x}}_{2} & \ldots & x_{n}\left(2\right)-{\bar{x}}_{n}\\ 0 & x_{1}\left(3\right)-{\bar{x}}_{1} & x_{2}\left(3\right)-{\bar{x}}_{2} & \ldots & x_{n}\left(3\right)-{\bar{x}}_{n}\\ \vdots & \vdots & \vdots \ddots & \vdots \\ 0 & x_{1}\left(K\right)-{\bar{x}}_{1} & x_{2}\left(K\right)-{\bar{x}}_{2} & \ldots & x_{n}\left(K\right)-{\bar{x}}_{n} \end{array}\right)\left(\begin{array}{c} f_{i}\\ a_{i1}\\ a_{i2}\\ \vdots \\ a_{in} \end{array}\right)=\left(\begin{array}{c} {\bar{\dot{x}}}_{i}\\ {\dot{x}}_{i}\left(2\right)-{\bar{\dot{x}}}_{i}\\ {\dot{x}}_{i}\left(3\right)-{\bar{\dot{x}}}_{i}\\ \vdots \\ {\dot{x}}_{i}\left(K\right)-{\bar{\dot{x}}}_{i} \end{array}\right) \end{array}$$

Denote by R the matrix

$$\left(\begin{array}{cccc} x_{1}\left(2\right)-{\bar{x}}_{1} & x_{2}\left(2\right)-{\bar{x}}_{2} & \ldots & x_{n}\left(2\right)-{\bar{x}}_{n}\\ \vdots & \vdots & \vdots \ddots & \vdots \\ x_{1}\left(K\right)-{\bar{x}}_{1} & x_{2}\left(K\right)-{\bar{x}}_{2} & \ldots & x_{n}\left(K\right)-{\bar{x}}_{n} \end{array}\right),$$

q the vector ${\left(x_{i}\left(2\right)-{\bar{\dot{x}}}_{i},\ldots ,x_{i}\left(K\right)-{\bar{\dot{x}}}_{i}\right)}^{T}$, and $a_{i}$ the row vector ${\left(a_{i1},\ldots ,a_{in}\right)}^{T}$. Then, $Ra_{i}=q$. The least square solution of $a_{i}$, ${\hat{a}}_{i}$, solves

$$\begin{array}{c} R^{T}R{\hat{a}}_{i}=R^{T}q. \end{array}$$

Note that $R^{T}R$ is KC, where $C=(c_{ij})$ is the sample covariance matrix. Thus,

(27) $$\begin{array}{c} \left(\begin{array}{c} {\hat{a}}_{i1}\\ {\hat{a}}_{i2}\\ \vdots \\ {\hat{a}}_{in} \end{array}\right)=C^{-1}\left(\begin{array}{c} c_{1,di}\\ c_{2,di}\\ \vdots \\ c_{n,di} \end{array}\right) \end{array}$$

where $c_{j,di}$ is the sample covariance between the series $\left\{x_{j}\left(k\right)\right\}$ and $\left\{\left(x_{i}\left(k+1\right)-x_{i}\left(k\right)\right)/\Delta t\right\}$.

Thus, finally, the mle of $T_{A\to B}$ is

(28) $$\begin{array}{c} {\hat{T}}_{A\to B}=\sum_{i=r+1}^{s}\left[\sum_{j=r+1}^{s}c_{ij}^{\prime }\left(\sum_{k=1}^{s}{\hat{a}}_{ik}c_{kj}\right)-{\hat{a}}_{ii}\right], \end{array}$$

where $c_{ij}^{\prime }$ is the (i,j)th entry of ${\tilde{C}}^{-1}$, and

(29) $$\begin{array}{c} \tilde{C}=\left(\begin{array}{cc} I_{r\times r} & 0_{r\times (s-r)}\\ 0_{(s-r)\times r} & \left[\begin{array}{ccc} c_{r+1,r+1} & \ldots & c_{r+1,s}\\ \vdots & \vdots & \vdots \\ c_{s,r+1} & \ldots & c_{s,s} \end{array}\right] \end{array}\right). \end{array}$$

Likewise,

(30) $$\begin{array}{ccc} {\hat{T}}_{B\to A} & = & \sum_{i=1}^{r}\left[\sum_{j=1}^{r}c_{ij}^{\prime \prime }\left(\sum_{k=1}^{s}{\hat{a}}_{ik}c_{kj}\right)-{\hat{a}}_{ii}\right] \end{array}$$

Here,

(31) $$\begin{array}{c} \tilde{\tilde{C}}=\left(\begin{array}{cc} \left[\begin{array}{ccc} c_{11} & \ldots & c_{1r}\\ \vdots & \vdots & \vdots \\ c_{r1} & \ldots & c_{rr} \end{array}\right] & 0_{r\times (s-r)}\\ 0_{(s-r)\times r} & I_{\left(s-r)\times (s-r\right)} \end{array}\right), \end{array}$$

and $c_{ij}^{\prime \prime }$ is the (i,j)th entry of ${\tilde{\tilde{C}}}^{{}_{-}1}$.

When n=2 and r=1, and hence, s=1, $\tilde{\tilde{C}}=\left[\begin{array}{cc} c_{11} & 0\\ 0 & 1 \end{array}\right]$, so $c_{11}^{\prime \prime }=c_{11}^{-1}$. Equation (30) thus becomes

$$\begin{array}{ccc} {\hat{T}}_{B\to A} & = & c_{11}^{\prime \prime }\sum_{k=1}^{2}{\hat{a}}_{1k}c_{k1}-{\hat{a}}_{11}\\ & = & \frac{1}{c_{11}}\left({\hat{a}}_{11}c_{11}+{\hat{a}}_{12}c_{12}\right)-{\hat{a}}_{11}\\ & = & \frac{c_{11}c_{12}c_{2,d1}-c_{12}^{2}c_{1,d1}}{c_{11}^{2}-c_{11}c_{12}^{2}}, \end{array}$$

recovering the well-known Equation (10) in [19].

## 4. Validation

### 4.1. One-Way Causal Relation

**Figure 1:**
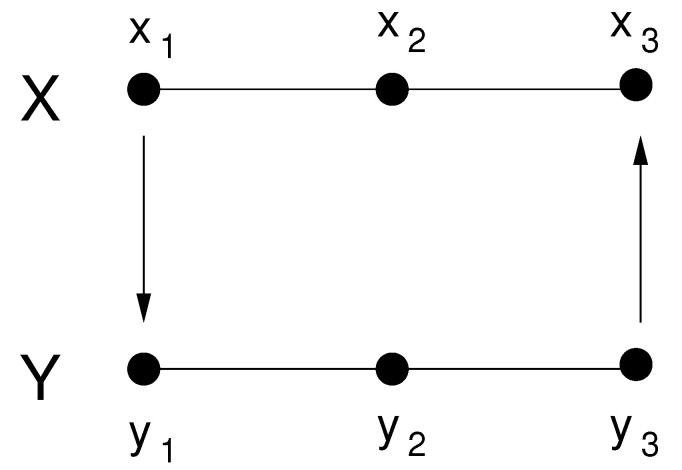
The preset coupling between the subsystems *X* and *Y*.

To see if the above formalism works, consider the vector autoregressive (VAR) process:

(32) $$\begin{array}{ccc} & & X:\;\left\{\begin{array}{c} x_{1}\left(n+1\right)=-0.5x_{1}\left(n\right)+0.5x_{2}\left(n\right)+0.2x_{3}\left(n\right)+e_{x1}\left(n+1\right),\\ x_{2}\left(n+1\right)=0x_{1}\left(n\right)-0.2x_{2}\left(n\right)-0.6x_{3}\left(n\right)+e_{x2}\left(n+1\right),\\ x_{3}\left(n+1\right)=-0.2x_{1}\left(n\right)+0.4x_{2}\left(n\right)-0.2x_{3}\left(n\right)+\varepsilon_{3}y_{3}\left(n\right)+e_{x3}\left(n+1\right), \end{array}\right. \end{array}$$

(33) $$\begin{array}{ccc} & & Y:\;\left\{\begin{array}{c} y_{1}\left(n+1\right)=-0.2y_{1}\left(n\right)-0.5y_{2}\left(n\right)+0y_{3}\left(n\right)-\varepsilon_{1}x_{1}\left(n\right)+e_{y1}\left(n+1\right),\\ y_{2}\left(n+1\right)=0.5y_{1}\left(n\right)-0.6y_{2}\left(n\right)+0.4y_{3}\left(n\right)+e_{y2}\left(n+1\right),\\ y_{3}\left(n+1\right)=-0.1y_{1}\left(n\right)-0.4y_{2}\left(n\right)-0.5y_{3}\left(n\right)+e_{y3}\left(n+1\right) \end{array}\right., \end{array}$$

where $e_{xi},e_{yi}∼N\left(0,1\right)$, i=1,2,3, are independent. As schematized in Figure 1, $\left(x_{1},x_{2},x_{3}\right)$ and $\left(y_{1},y_{2},y_{3}\right)$ form two subsystems, written as *X* and *Y*, respectively. They are coupled only through the first and third components; more specifically, $x_{1}$ drives $y_{1}$, and *Y* feeds back to *X* through coupling $y_{3}$ with $x_{3}$. The strength of the coupling is determined by the parameters $\varepsilon_{1}$ and $\varepsilon_{3}$. In this subsection, $\varepsilon_{3}=0$, so the causality is one-way, i.e., from *X* to *Y* without feedback.

**Figure 2:**
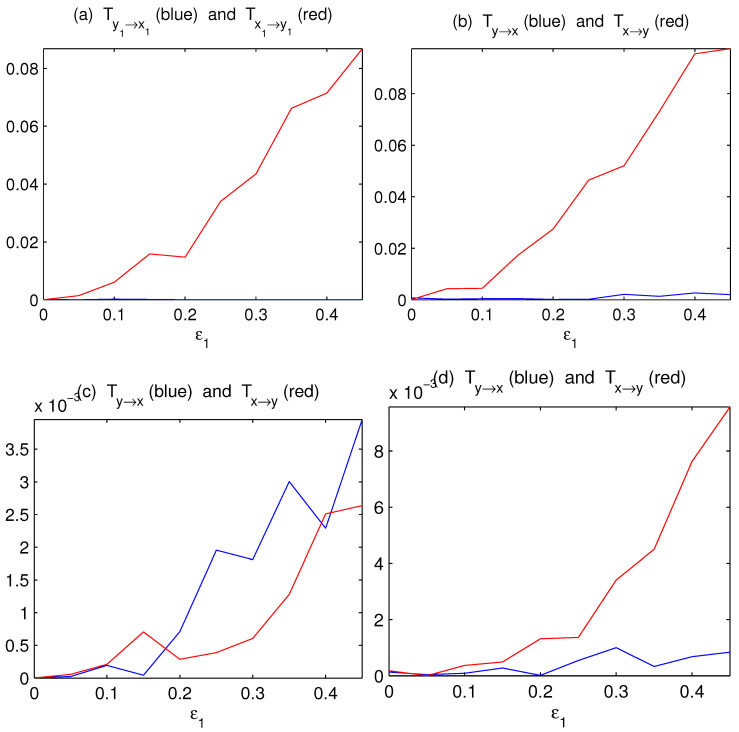
The absolute information flows between subspaces *X* and *Y* as functions of the coupling coefficients $\varepsilon_{1}$ ($\varepsilon_{3}=0$). (**a**) The componentwise information flows between $x_{1}$ and $y_{1}$; (**b**) the bulk information flows between subsystems *X* and *Y* computed with Equations (28) and (30); (**c**) the information flows between $\bar{x}$ and $\bar{y}$; (**d**) the information flows between the first principal components of ($x_{1},x_{2},x_{3}$) and ($y_{1},y_{2},y_{3}$), respectively (units: nats per time step).

Initialized with random numbers, we iterate the process for 20,000 steps and discard the first 10,000 steps to form six time series with a length of 10,000 steps. Using the algorithm by Liang (e.g., [16,18,19,20]), the information flows between $x_{1}$ and $y_{1}$ can be rather accurately obtained. As shown in Figure 2a, the information flow/causality from *X* to *Y* increases with $\varepsilon_{1}$, and there is no causality the other way around, just as expected. Since no other coupling exists, one can imagine that the bulk information flows must also bear a similar trend. Using Equations (28) and (30), the estimators are indeed similar to that, as shown in Figure 2b. This demonstrates the success of the above formalism.

Since practically averages and principal components (PCs) have been widely used to measure complex subsystem variations, we also compute the information flows between $\bar{x}=\frac{1}{3}\left(x_{1}+x_{2}+x_{3}\right)$ and $\bar{y}=\frac{1}{3}\left(y_{1}+y_{2}+y_{3}\right)$, and that between the first PCs of $\left(x_{1},x_{2},x_{3}\right)$ and $\left(y_{1},y_{2},y_{3}\right)$. The results are plotted in Figure 2c,d, respectively. As can be seen, the principal component analysis (PCA) method works just fine in this case. By comparison, the averaging method yields an incorrect result.

The incorrect inference based on averaging is within expectation. In a network with complex causal relations, for example, with a causality from $y_{2}$ to $y_{1}$, the averaging of $y_{1}$ with $y_{2}$ is equivalent to mixing $y_{1}$ with its future state, which is related to the contemporary state of $x_{1}$, and hence will yield a spurious causality to $x_{1}$. The PCA here functions satisfactorily, perhaps because in selecting the most coherent structure, it discards most of the influences from other (implicit) time steps. However, the relative success of PCA may not be robust, as evidenced in the following mutually causal case.

### 4.2. Mutually Causal Relation

**Figure 3:**
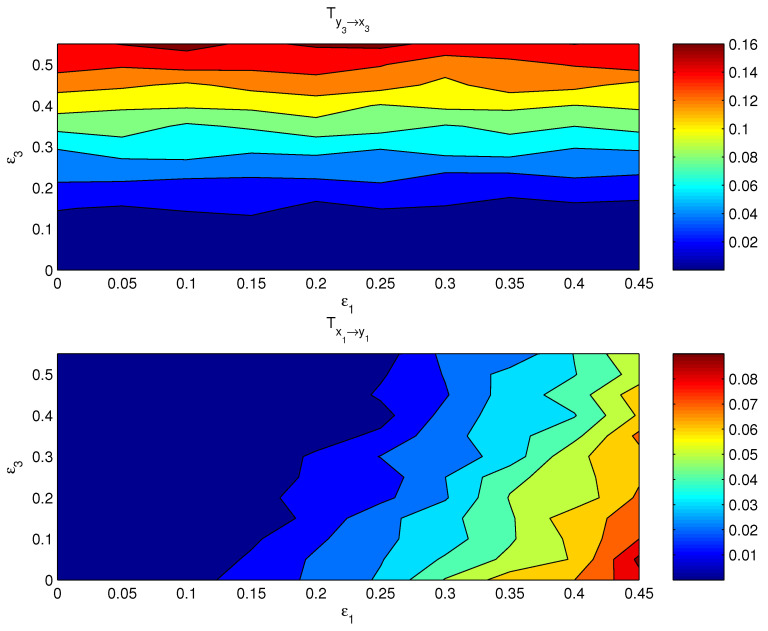
The absolute information flow from $y_{3}$ to $x_{3}$ and that from $x_{1}$ to $y_{1}$ as functions of $\varepsilon_{1}$ and $\varepsilon_{3}$. The units are in nats per time step.

**Figure 4:**
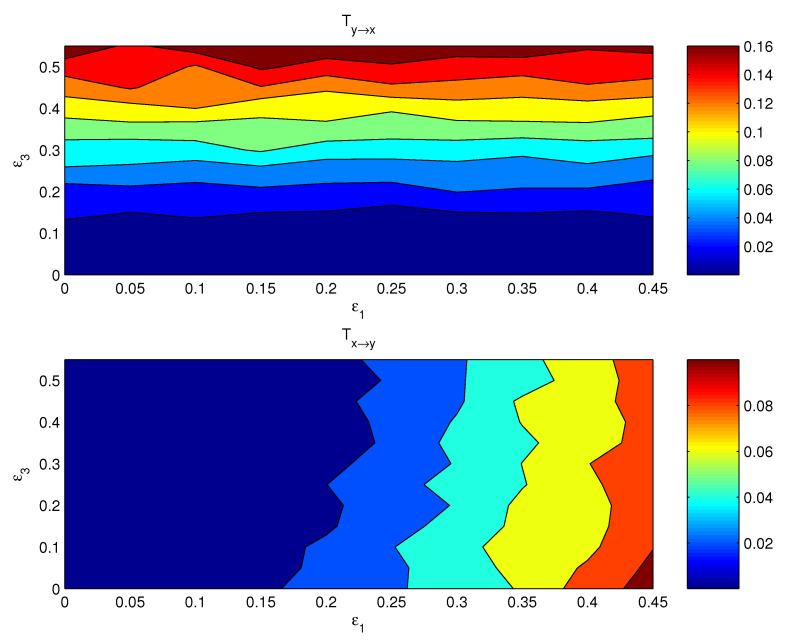
The absolute bulk information flow from subsystem *Y* to subsystem *X*, and that from *X* to *Y*. The abscissa and ordinate are the coupling coefficients $\varepsilon_{1}$ and $\varepsilon_{3}$, respectively.

If both the coupling parameters, $\varepsilon_{1}$ and $\varepsilon_{3}$, are turned on, the resulting causal relation has a distribution on the $\varepsilon_{1}-\varepsilon_{3}$ plane. Figure 3 shows the componentwise information flows $T_{x_{1}\to y_{1}}$ (bottom) and $T_{y_{3}\to x_{3}}$ (top) on the plane. The other two flows, i.e., their counterparts $T_{y_{1}\to x_{1}}$ and $T_{x_{3}\to y_{3}}$, are by computation essentially zero. As argued in the preceding subsection, the bulk information flows should follow the general pattern, albeit perhaps in a more coarse and/or mild pattern, since it is a property on the whole. This is indeed true. Shown in Figure 4 are the bulk information flows between *X* and *Y* computed using Equations (28) and (30).

**Figure 5:**
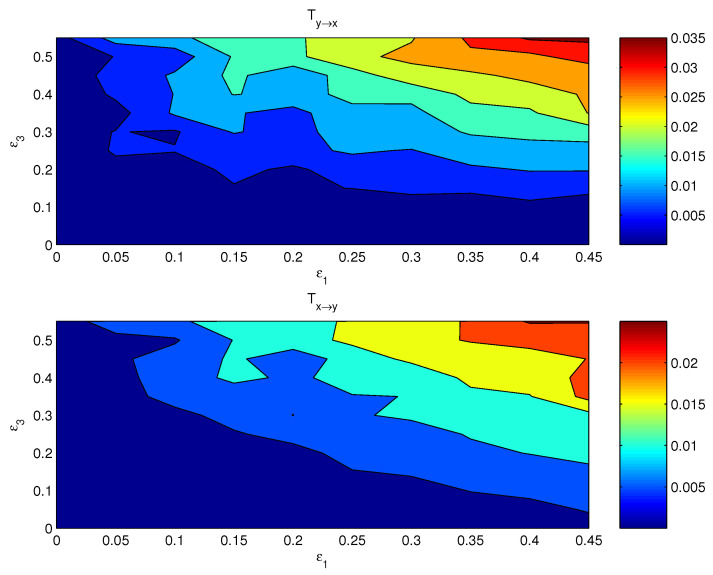
As Figure 4, but for the information flows between the mean series $\bar{x}=\frac{1}{3}\left(x_{1}+x_{2}+x_{3}\right)$ and $\bar{y}=\frac{1}{3}\left(y_{1}+y_{2}+y_{3}\right)$.

Again, as usual, we try the averages and first PCs as proxies for estimating the causal interaction between *X* and *Y*. Figure 5 shows the distributions of the information flows between $\bar{x}$ and $\bar{y}$. The resulting patterns are totally different from what Figure 3 displays; obviously, these patterns are incorrect.

**Figure 6:**
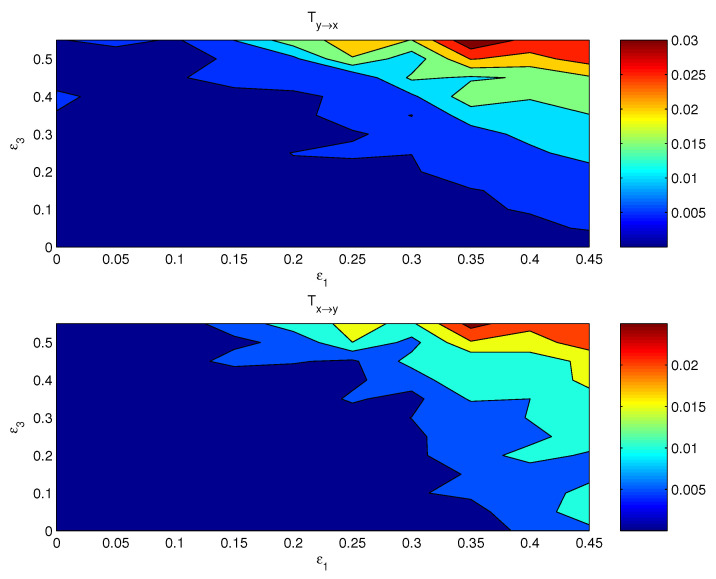
As Figure 4, but for the information flows between the first principal component of $\left(x_{1},x_{2},x_{3}\right)$ and that of $\left(y_{1},y_{2},y_{3}\right)$.

One may expect that the PCA method should yield more reasonable causal patterns. We have computed the first PCs for $\left(x_{1},x_{2},x_{3}\right)$ and $\left(y_{1},y_{2},y_{3}\right)$, respectively, and estimated the information flows using the algorithm by Liang [20]. The resulting distributions, however, are no better than those with the averaged series (Figure 6). That is to say, this seemingly more sophisticated approach does not yield the right interaction between the complex subsystems, either.

## 5. Summary

Information flow provides a natural measure of the causal interaction between dynamical events. In this study, the information flows between two complex subsystems of a large dimensional system are studied, and analytical formulas have been obtained in a closed form. For easy reference, the major results are summarized hereafter.

For an *n*-dimensional

$$\begin{array}{c} \frac{dx}{dt}=F\left(x,t\right)+B\left(x,t\right)\dot{w}, \end{array}$$

if the probability density function (pdf) of x is compactly supported, then the information flows from subsystem *A*, which are made of $x_{1\ldots r}$, to subsystem *B*, made of $x_{r+1,\ldots ,s}$ (1≤r<s≤n), and that from *B* to *A* are, respectively (in nats per unit time),

$$\begin{array}{ccc} & & T_{A\to B}=-E\left[\sum_{i=r+1}^{s}\frac{1}{\rho_{r+1,\ldots ,s}}\int_{{\mathbb{R}}^{n-s}}\frac{\partial F_{i}\rho_{r+1,\ldots ,n}}{\partial x_{i}}dx_{s+1,\ldots ,n}\right]\\ & & \;\;\;\;\;+\frac{1}{2}E\left[\sum_{i=r+1}^{s}\sum_{j=r+1}^{s}\frac{1}{\rho_{r+1,\ldots ,s}}\int_{{\mathbb{R}}^{n-s}}\frac{\partial^{2}g_{ij}\rho_{r+1,\ldots ,n}}{\partial x_{i}\partial x_{j}}dx_{s+1,\ldots ,n}\right], \end{array}$$

$$\begin{array}{ccc} & & T_{B\to A}=-E\left[\sum_{i=1}^{r}\frac{1}{\rho_{1\ldots r}}\int_{{\mathbb{R}}^{n-s}}\frac{\partial F_{i}\rho_{1,\ldots ,r,s+1,\ldots ,n}}{\partial x_{i}}dx_{s+1,\ldots ,n}\right]\\ & & \;\;\;\;\;+\frac{1}{2}E\left[\sum_{i=1}^{r}\sum_{j=1}^{r}\frac{1}{\rho_{1\ldots r}}\int_{{\mathbb{R}}^{n-s}}\frac{\partial^{2}g_{ij}\rho_{1,\ldots ,r,s+1,\ldots ,n}}{\partial x_{i}\partial x_{j}}dx_{s+1,\ldots ,n}\right], \end{array}$$

where $g_{ij}=\sum_{k=1}^{m}b_{ik}b_{jk}$, and *E* signifies mathematical expectation. Given *n* stationary time series, $T_{A\to B}$ and $T_{B\to A}$ can be estimated. The maximum likelihood estimators under a Gaussian assumption are referred to in Equations (28) and (30).

We have constructed a VAR process to validate the formalism. The system has a dimension of 6, with two subsystems respectively denoted by *X* and *Y*, each with a dimension of 3. *X* drives *Y* via the coupling at one component, and *Y* feeds back to *X* via another. The detailed, componentwise causal relation can be easily found using our previous algorithms such as that in [20]. It is expected that the bulk information flow should in general also follow a similar trend, though the structure could be in a more coarse and mild fashion, as now displayed is an overall property. The above formalism does yield such a result. On the contrary, the commonly used proxies for subsystems, such as averages and principal components (PCs), generally do not work. Particularly, the averaged series yield the wrong results in the two cases considered in this study; the PC series do not work either for the mutually causal case, though they result in a satisfactory characterization for the case with a one-way causality.

The result of this study is applicable in many real world problems. As explained in the Introduction, it will be of particular use in the related fields of climate science, neuroscience, financial economics, fluid mechanics, etc. For example, it helps clarify the role of greenhouse gas emissions in bridging the climate system and the socioeconomic system (see a review in [26]). Likewise, the interaction between the earth system and public health [27] can also be studied. In short, it is expected to play a role in the frontier field of complexity, namely, multiplex networks or networks of networks (see the references in [28,29,30]). We are therefore working on these applications.
